# Outcome Measures in Tele-Rehabilitation and Virtual Reality for Stroke Survivors: Protocol for a Scoping Review

**DOI:** 10.5539/gjhs.v8n1p79

**Published:** 2015-05-15

**Authors:** Mirella Veras, Dahlia Kairy, Marco Rogante, Claudia Giacomozzi

**Affiliations:** 1 École de réadaptation, Faculté de Médecine, Université de Montréal; CRIR site Institut de réadaptation Gingras-Lindsay de Montréal, Montreal, Quebec, Canada; 2 Dipartimento Tecnologie e Salute, Istituto Superiore di Sanità, Roma, Italy

**Keywords:** stroke, virtual reality, outcome measures, telerehabilitation, scoping review

## Abstract

Despite the increased interest about tele-rehabilitation, virtual reality and outcome measures for stroke rehabilitation, surprisingly little research has been done to map and summarize the most common outcome measures used in tele-rehabilitation. For this review, we propose to conduct a systematic search of the literature that reports outcome measures used in tele-rehabilitation or virtual reality for stroke rehabilitation. Specific objectives include: 1) to identify the outcome measures used in tele-rehabilitation studies; 2) to describe the psychometric properties of the outcome measures in the included studies; 3) to describe which parts of the International Classification of Functioning are measured in the studies. Methods: we will conduct a comprehensive search of relevant electronic databases (e.g., PUBMED, CINAHL, EMBASE, PSYCOINFO, Cochrane Central Register of Controlled Trial and PEDRO). The scoping review will include all study designs. Two reviewers will pilot-test the data extraction forms and will independently screen all the studies and extract the data. Disagreements about inclusion or exclusion will be resolved by consensus or by consulting a third reviewer. The results will be synthesized and reported considering the implications of the findings within the clinical practice and policy context. Dissemination: we anticipate that this scoping review will contribute to inform researchers and end-users (ie, clinicians and policy-makers), regarding the most appropriate outcome measures for tele-rehabilitation or virtual reality as well as help to identify gaps in current measures. Results will be disseminated through reports and open access journals, conference presentations, as well as newsletters, podcasts and meetings targeting all the relevant stakeholders.

## 1. Introduction

Tele-rehabilitation (TR) and virtual reality (VR) are alternative and innovative approaches of delivering rehabilitation services for stroke survivors (Laver et al., 2013; Laver, George, Thomas, Deutsch, & Crotty, 2011). Tele-rehabilitation is defined as “the provision of rehabilitation services to patients at a remote location using information and communication technologies” (Brennan, Mawson, & Brownsell, 2009). Virtual reality, in its turn, is defined as the “use of interactive simulations created with computer hardware and software to present users with opportunities to engage in environments that appear and feel similar to real-world objects and events” (Weiss, Kizony, Feintuch, & Katz, 2006). The communication between the patient and the rehabilitation professional is made possible using technologies such as telephone, videoconferencing through internet and sensors (Brennan, Mawson, & Brownsell, 2009). Measuring the effectiveness of these interventions is crucial to assess their effectiveness and the development of evidence in tele-rehabilitation and virtual reality.

Several tools have been developed to evaluate the consequences of stroke and the effectiveness of virtual reality

and telerehabilitation interventions on stroke outcomes (Salter, Foley, Jutai, & Teasell, 2007). Evidence-based review in stroke rehabilitation has identified numerous assessment tools used in stroke (Canadian Stroke Network, 2014). Another review of outcome measures in stroke rehabilitation used in Randomized Controlled Trials (RCTs) identified only 30 measures reported in RCTs that examined the efficiency of stroke rehabilitation interventions (Salter, Foley, Jutai, & Teasell, 2007). Rehabilitation services for stroke survivors could be enhanced by use of standardized outcomes (Canadian Stroke Network, 2014). It is recognized that their use to clinically examine patient improvement should consider not only impairments in body function, but also different aspects of patients’ life participation, daily activity preferences and believes (Jette, Halbert, Iverson, Miceli, & Shah, 2009). The benefits of using standardized outcome measures include identifying patients who are at risk for poor or adverse outcomes (Weiss, Kizony, Feintuch, & Katz, 2006), determining the most-effective interventions in specific contexts and assessing organizational performance (Weiss, Kizony, Feintuch, & Katz, 2006). Although the use of standardized instruments in rehabilitation has been advocated for use by clinicians for several years, there is no consensus about the use of outcome measures to facilitate comparisons across interventions and studies (Jette, Halbert, Iverson, Miceli, & Shah, 2009). To our knowledge there are only reviews dealing with efficacy/efficiency of TR or VR as well as reviews on outcome measures for rehabilitation. This review will likely be the first one to address outcome measures used in VR and TR. Former considerations are also applicable to rehabilitation delivered remotely. In order to provide guidelines for the use of outcome measures in tele-rehabilitation and virtual reality in the field of stroke rehabilitation, a scoping review will be conducted and will provide a synthesis of the most used and appropriate outcome measures. The present paper aims at describing the protocol for the scoping review.

## 2. Method

Former systematic reviews on tele-rehabilitation and virtual reality addressed the difficulties of finding evidence coming from the small number of eligible studies. Scoping reviews may overcome this problem since their approach may address other questions beyond those related to intervention effectiveness, and generate findings that can complement the findings of clinical trials.

The scoping review will use a methodologically rigorous approach, guided by the Arksey and O’Malley framework from the University of York (Arksey & O’Malley, 2005). The York framework has been used extensively in knowledge synthesis studies (Kastner et al., 2012; Aarts et al., 2012) and has five stages as follows: stage 1) Identifying the research question; stage 2) Identifying relevant studies according to research question; stage 3) Study selection; stage 4) Charting the data within the selected studies and stage 5) Collating, summarizing and reporting the results of the scoping review. The research questions for this review are:

- Which tele-rehabilitation and virtual reality outcome measures are used and when are they administrated (admission, discharge and follow-up of the patient) following a stroke?
- Which measures are psychometrically sound and what are the author(s) trying to measure? (Change to patient, to health professional, to organization/service or minimal clinically important difference).
- Which parts of the International Classification of Functioning are measured in the outcome measures? (Body functions (consciousness functions, orientation functions, muscle power functions mental functions of language, attention functions, memory functions) and structures (structure of brain, structure of upper extremity, structure of lower extremity), activities and participation (walking, speaking, toileting, eating, dressing, communicating-receiving, spoken messages) and environmental factors (immediate family, health care providers, health care system).

A possible limitation of scoping reviews is the quality of the included studies since generally it’s not assessed; to overcome this limitation a quality assessment will be conducted in parallel to the stages of the scoping review, which will be based on the CASP tools (CAPS, 2015). Results of this activity will be qualitatively summarized and added to the main findings of the study

### 2.1 Eligibility Criteria

The scoping review will include studies: 1) involving stroke patients; 2) describing a rehabilitation intervention using tele-rehabilitation and/or virtual reality; 3) written in English, French, Spanish, Italian or Portuguese. The exclusion criteria are: articles published in other languages; articles not reporting the outcome measures or only reporting laboratory measures or only reporting the development of the technology. The search will not be limited by study design. Two authors will be in charge for the assessment of the retrieved papers; disagreement about inclusion or exclusion of a specific paper based on the review of its abstract will be resolved by reaching a motivated consensus or consulting a third reviewer.

### 2.2 Search Strategy

The literature search will be performed by a librarian expert in the field of rehabilitation. The search will include the major databases in which rehabilitation articles are found: PubMed (until December 2014), EMBASE (until December 2014), Cochrane Library-The Cochrane Central Register of Controlled Trials (until January 2015), Cumulative Index to Nursing and Allied Health Literature (CINAHL) (until December 2014) and PEDRO (until January 2015) to identify potentially relevant records. A combination of medical sub-headings (MeSH) and or keywords will be adapted as needed for each database (the details of the search strategy are available upon request).

Data collection process and charting the data within the selected studies

Data-exstraction forms will be developed based on the current literature in the field and on the research questions. A pilot test will be conducted using a subset of articles covering the research questions; changes may be necessary in the data extraction form during this validation phase in order to reach a final version. We expect to collect data on: 1) articles’ authors, 2) year of publication, 3) objective(s) of the study, 5) study design, 6) country 7) outcome measures reported; 8) psychometric properties of measures reported, and 9) participants’ characteristics (e.g. age, gender, socio-economic status, level of education, functional level, stroke phase, type of stroke); 10) relevant International Classification of Functioning domains.; 11) period of time at which the measure is taken (e.g. admission, discharge, follow-up); 12) technology used (virtual reality, tele-rehabilitation, both); 13) details on tele-rehabilitation and/or virtual reality intervention and 14) outcome improvement.

The outcome measures will be organized according to the domains of the international classification of functioning.

### 2.3 Synthesis

Based on the recommendations by Levac et al. (2010), data synthesis will rely on: i) a numerical summary and qualitative thematic analysis; ii) results organized and summarized quantitatively in tables and described qualitatively; iii) considerations on the implications of study findings to policy, practice, or research. Furthermore main results of the quality assessment will be qualitatively summarized and added to the main findings of the study. The results will help identify the most used outcome measures whether or not they show a significant effect. It will also give us information on what these measures will allow us to document for tele-rehabilitation and virtual reality in the field of stroke as well as the gaps in knowledge that require future research, including a systematic review.

## 4. Discussion and Conclusion

The results of the proposed scoping review are addressed primarily to clinicians and researchers in rehabilitation. In addition, the results will be disseminated to a larger audience including clinicians, policy makers, researchers and stakeholders and those interested in standardizing outcome measures for stroke tele-rehabilitation and virtual reality. Our research team will ensure broad dissemination of our findings through numerous knowledge translation strategies including podcasts, publications in open-access and peer-reviewed journals, conference presentations, web seminars and meetings including diverse audiences such as clinicians, stakeholders, researchers, healthcare managers, policy-makers and citizens, among others.
